# International inter-school competition to encourage children to walk to school: a mixed methods feasibility study

**DOI:** 10.1186/s13104-014-0959-x

**Published:** 2015-01-27

**Authors:** Ruth F Hunter, Debra de Silva, Veronica Reynolds, William Bird, Kenneth R Fox

**Affiliations:** Centre for Public Health, Queen’s University Belfast, Belfast, Northern Ireland UK; UKCRC Centre of Excellence for Public Health (NI), Queen’s University Belfast, Belfast, Northern Ireland UK; The Evidence Centre, London, England UK; Intelligent Health Ltd, Reading, England UK; Centre for Exercise, Nutrition and Health Sciences, University of Bristol, Bristol, England UK

**Keywords:** Children, Physical activity, Active travel, Walking, Competition, International, Novel technology, Schools, Mixed methods, Feasibility study

## Abstract

**Background:**

Active travel to school can be an important contributor to the total physical activity of children but levels have declined and more novel approaches are required to stimulate this as an habitual behaviour. The aim of this mixed methods study was to investigate the feasibility of an international walk to school competition supported by novel swipecard technology to increase children’s walking to/from school.

**Methods:**

Children aged 9–13 years old participated in an international walk to school competition to win points for themselves, their school and their country over a 4-week period. Walks to and from school were recorded using swipecard technology and a bespoke website. For each point earned by participants, 1 pence (£0.01) was donated to the charity of the school’s choice. The primary outcome was number of walks to/from school objectively recorded using the swipecard tracking system over the intervention period. Other measures included attitudes towards walking collected at baseline and week 4 (post-intervention). A qualitative sub-study involving focus groups with children, parents and teachers provided further insight.

**Results:**

A total of 3817 children (mean age 11.5 ± SD 0.7) from 12 schools in three cities (London and Reading, England and Vancouver, Canada) took part in the intervention, representing a 95% intervention participation rate. Results show a gradual decline in the average number of children walking to and from school over the 4-week period (week 1 mean 29% ± SD2.5; week 2 mean 18% ± SD3.6; week 3 mean 14% ± SD4.0; week 4 mean 12% ± SD1.1). Post intervention, 97% of children felt that walking to school helped them stay healthy, feel happy (81%) and stay alert in class (76%). These results are supported by qualitative findings from children, parents and teachers. Key areas for improvement include the need to incorporate strategies for maintenance of behaviour change into the intervention and also to adopt novel methods of data collection to increase follow-up rates.

**Conclusions:**

This mixed methods study suggests that an international walk to school competition using innovative technology can be feasibly implemented and offers a novel way of engaging schools and motivating children to walk to school.

**Electronic supplementary material:**

The online version of this article (doi:10.1186/s13104-014-0959-x) contains supplementary material, which is available to authorized users.

## Background

The prevalence of physical inactivity, obesity and associated health conditions in childhood is rising [1]. Recent guidance from the UK Chief Medical Officer’s (CMO), recommends that children aged 5–18 years old should engage in at least 60 minutes per day of moderate-vigorous physical activity to receive health benefits [2]. A recent study reported that only 24% of children in England aged 5–15 years met these recommendations, which is significantly lower in girls (19%) than boys (29%) [3]. Further, physical activity in adolescence declines by 7% per year [4], suggesting an overall decline of 60-70% during the 10–19 years old period [5]. Physical activity habits formed at this crucial time can often be lifelong and could have potentially long-term benefits.

Active travel to school, such as walking and cycling, can be an important contributor to the total physical activity of children [6]. Its regular nature helps it become a habitual behaviour that has potential to track into adulthood [7]. However, levels are significantly declining in favour of drop off by car [8], with approximately 25% of ‘rush hour’ traffic attributable to the school run [9]. This in turn increases traffic congestion and pollution. Figures for the UK show that only 5 to 8% of total physical activity is attributable to active travel [3]. This is also an international problem, evident in other countries such as the United States, Canada and Australia [10-12] and merits broader attention. Developing effective and sustainable interventions to increase physical activity long-term and increasing active travel have therefore been highlighted as top research priorities for children and adolescent physical activity [13].

There is a plethora of research investigating the correlates of active travel in children [14-16], and promising findings from a recent study showed that a change to an active mode of travel to school was associated with an increase in daily minutes of overall activity [17]. However, previous reviews demonstrate that only a limited number of interventions have been found effective for increasing children’s physical activity [6,18-20] which suggests a need for novel approaches, in particular to encourage walking and cycling to school.

Incentives to promote long-term healthy behaviour changes have been targeted as a priority by UK government for promoting public health [21]. Recent research from the behavioural economics literature has shown that competition (an extrinsic motivator) can act as an effective incentive for stimulating increases in physical activity in adults and children [22,23]. Further, international competition involving multiple countries may increase interest and participation. However, there is a dearth of research in this area and the feasibility of implementing such an intervention is unknown.

Therefore, this mixed methods study investigated the feasibility of implementing an international walk to school competition to increase children’s physical activity levels. Objectives of the evaluation included determination of:

1. Effectiveness of school recruitment;
2. Effectiveness of recruitment of children for the intervention and evaluation;
3. Retention of children, including burden and success of data collection methods;
4. Preliminary evidence of potential of intervention to increase walk to/from school behaviour;
5. Areas for programme modification and improvement.

## Methods

### Study design

The study was an uncontrolled pre- and post- mixed methods evaluation of the feasibility of a 4-week international walk to school competition. The study was approved by the Research Ethics Committee of the School of Medicine, Dentistry and Biomedical Sciences, Queen’s University Belfast, Northern Ireland (Ref 12/27). This approval included agreement for the research team to analyse data collected from all schools. Appropriate approval from local authorities in all participating cities was gained prior to the start of the study. All participants and their parent/guardian provided fully informed written consent prior to participating in the intervention evaluation.

### Setting

The study involved schools (both primary and secondary level and their equivalents) based in major cities in England and Canada. It was also hypothesised that engaging children in the lives of those from other countries would add interest, stimulus and an educational element. In addition, including primary and secondary schools, and different countries facilitated investigation of the feasibility and acceptability of the intervention in a range of settings. The intervention was run simultaneously across all cities and schools in September/October.

### Recruitment

#### Schools

A sample of schools was selected to take part based on two main criteria: (1) local authorities had suggested an issue that could be addressed by the intervention, for example, high traffic congestion when parents drop children off near the school gates, low overall rates of physical activity or wanting to build greater social cohesion within the school; and (2) schools’ expressed willingness to participate. Members of the project team contacted local authorities and transport organisations in the participating cities to provide information about the proposed scheme. These organisations helped identify relevant personnel in schools. Schools expressing interest were contacted by a member of the project team to provide them with more information about the study.

#### Children

All children in years 7 and 8 (aged 11–13 years old) from each of the participating schools in England (secondary schools) and aged 9–12 years old in Canada (primary schools), were invited to take part. Members of the project team visited the school and gave a presentation during Assembly (i.e. regular gathering of students and teachers at the beginning of the school day). This was followed by a letter to all eligible children and their parents, which provided further information about the study and invited the child to take part. The letter provided contact details for the project team, and children and/or parents were encouraged to contact a member of the team to ask any questions. Interested children, their parents or guardians, provided written informed consent prior to taking part.

### Intervention

The complex intervention (known as “Beat the Street”), underpinned by Learning Theory [24] and Social Cognitive Theory [25], involved several interacting components including an international walk to school competition, incentives (retail vouchers, charity donations), novel technology (involving Near Field Communication and Radio Frequency Identification tags), and a bespoke website. The intervention was implemented by Intelligent Health Ltd, a health IT company who develop and implement physical activity programmes.

#### Competition

Children competed in an international walk to school competition to win points for themselves, their school and their country over a 4-week competition period. Children were awarded two points for trips to/from school up to 0.5 km and three points for trips greater than 0.5 km. Only trips to and/or from school were monitored and awarded points. These buffers were based on the average distance that children travel to school [26]. Participants also received two points for participating and reporting data at follow-up time points. For each point earned by participants one pence (£0.01) was donated to the charity of the school’s choice. Prizes (12 in total over the competition period) were awarded randomly by a member of the project team once a week to participants which included £10 vouchers for a local retailer (three vouchers per week) and a family day out to a theme park. The schools also rewarded their top 10 performers with their own in-house rewards, for example, certificates which were awarded by the Head teacher.

#### Swipecard technology

Walks to and from school were monitored and recorded using novel technology and a bespoke website. Children scanned a swipecard across sensors placed along walking routes to log their walking behaviour to and from school. Sensors were attached to lampposts at public transport links and school gates marking walking routes around 1 km in length (see Additional file 1). The position of the sensors was selected in collaboration with the local authority to encourage use of feasible routes in high catchment areas. When children swiped their cards on a sensor this created a timestamp (logging date and time of walk), and the information was automatically sent to an online system. Children who lived within 1–2 km walking distance to school were encouraged to walk the entire trip and those who lived further away were encouraged to start their walk from a sensor by getting off the bus a stop earlier or ask their parents to stop the car a few streets away so they could walk the rest of the journey to school.

#### Website

A bespoke website served several functions including providing feedback on walking behaviour and monitoring progress in the competition using a league table format. Children could monitor group-level scores online. The website also contained several educational and motivational tools such as maps of sensor locations and walking routes, and encouraged peer support through online message boards which were moderated daily by the project team.

### Measures

Demographic characteristics including age, gender, school attended and usual mode of school travel were collected at baseline. School type (mixed, single gender) and size (number of students) were recorded. The primary outcome was number of walks to/from school objectively measured using the swipecard technology system during each day of the 4-week intervention. A bout of walking to or from school was recorded by a participant touching their swipecard on at least two sensors (i.e. at the bus stop and then at the school gate). A similar method was employed in a previous study to objectively monitor and infer physical activity levels from data collected routinely from the swipecards [27].

Other measures included a 5-day diary which recorded mode of travel to and from school and duration of journey (in minutes). Attitudes towards walking, active travel and social aspects of physical activity were also collected using Likert scales and multi-choice questions. These measures were collected at baseline and week 4 (immediate post-intervention) via the project website or paper-based questionnaires. Using similar methods, an online survey was completed post-intervention by teachers and parents to capture their attitudes towards children taking part in the competition and perceived changes in children’s physical activity.

#### Qualitative evaluation

A qualitative sub-study post-intervention involved focus groups with eight schools in London (n = 8 focus groups), Reading (n = 6 focus groups) and Vancouver (n = 2 focus groups) (16 focus groups/320 children), semi-structured follow-up telephone interviews with parents (n = 30) and teachers (n = 30) to explore the feasibility and potential benefits and challenges of the competition. Topics included feasibility of the swipecard technology as an evaluation method and motivational strategy and acceptability of the data collection methods. Participants were selected to provide diversity in age and gender. Interviews were audio-recorded and transcribed verbatim.

### Analyses

Recruitment rate was assessed by collating the actual number of children recruited versus the number invited to participate in the intervention and evaluation. Retention rates of children were measured as the proportion lost to follow-up post-intervention (week 4). Proportion of walks to/from school (based on number of participants registered per school) was aggregated for each week of the intervention for each school. Proportion of children using each mode of travel was derived from the 5-day travel diary at baseline.

Mean and 95% CI were calculated for the primary outcome for each week of the intervention. As this was a feasibility study, significance tests for change were not performed. Frequencies and cross-tabulations were calculated for attitudes towards walking, active travel and social aspects of physical activity at baseline and post-intervention. Data were analysed using the Statistical Package for Social Sciences (SPSS) version 18.0 (SPSS Inc, Chicago, US).

Transcripts of the focus group discussions and semi-structured interviews were read repeatedly and coded, using QSR NVivo 8 software (QSR International Inc, Massachusetts, US). Thematic Content Analysis [28] produced themes relating to perspectives of children, parents and teachers on the issues covered and help point to design and implementation modifications for further development and testing.

## Results

### School recruitment (objective 1)

Table 1 details the characteristics of the participating schools. Recruitment of the schools took 1-month duration and included schools from Vancouver, Canada (n = 2); London, England (n = 7) and Reading, England (n = 3). In Canada, the two schools selected were primary schools and in England, the schools were large secondary schools. The main reasons provided by schools for participating were reducing traffic congestion and increasing physical activity levels. Two schools in England were excluded from participating due to failure to acquire permission to install the technology on local streets in the required time-frame or the school wanting to change the competition format.

**Table 1:** **Characteristics of participating schools**

**City**	**School**	**Type**	**School size (total no. of students)**	*No. of participants (% of total sample)	**Reason for participation**
**London**	**L 1**	Secondary; 11–14 yrs	1500	600 (16%)	Increase physical activity; reduce traffic congestion
	**L 2**	Secondary; 11–14 yrs; all boys	625	250 (6%)	Increase physical activity; reduce traffic congestion
	**L 3**	Secondary; 11–14 yrs	800	317 (8%)	Reduce traffic congestion; increase road safety
	**L 4**	Secondary; 11–14 yrs	600	240 (6%)	Increase physical activity; increase social cohesion
	**L 5**	Secondary; 11–14 yrs	750	310 (8%)	Increase social cohesion; increase use of open spaces; reduce traffic congestion
	**L6**	Secondary; 11–14 yrs	750	310 (8%)	Increase social cohesion; increase use of open spaces; reduce traffic congestion
	**L7**	Secondary; 11–14 yrs; all girls	1200	480 (13%)	Reduce traffic congestion; increase physical activity
**Summary**			Mean 889	Total 2507	
**Reading**	**R 1**	Secondary; 11–14 yrs	1100	440 (12%)	Increase physical activity; improve links with international schools
	**R 2**	Secondary; 11–14 yrs	750	300 (8%)	Reduce traffic congestion; increase physical activity
	**R 3**	Secondary; 11–14 yrs; all girls	630	252 (7%)	Reduce traffic congestion; increase physical activity; increase road safety
**Summary**			Mean 827	Total 992	
**Vancouver**	**V 1**	Primary; 5–13 yrs	218	206 (5%)	Reduce traffic congestion; decrease car transport to school
	**V 2**	Primary; 5–13 yrs	132	112 (3%)	Reduce traffic congestion; decrease car transport to school
**Summary**			Mean 175	Total 318	
**Overall**	-	-	-	Total 3817	-

*Number who agreed to participate in the intervention.

### Recruitment of children (objective 2)

In total, 4,009 children from 12 participating schools were invited to take part. Of these, 95% (n = 3817) agreed to participate in the intervention; n = 318 (8%) from Vancouver; n = 2507 (66%) from London, and n = 992 (26%) from Reading). All children were recruited over a 2-month period (August-September). Table 2 details the characteristics of the participating children. Children were aged 9–13 years old (mean 11.5 ± SD 0.7) and 55% (n = 1145) were female. Overall, the majority of children were White (n = 862/50%), 13% (n = 224) were Asian, 8% (n = 132) were Black, and 29% (n = 493) identified with other ethnic groups (NB: Data regarding ethnicity were not collected from schools in Vancouver, Canada). The majority of children (n = 1515/73%) at baseline indicated that their usual mode of travel to school involved at least some walking (either all or part of the journey), with 5% (n = 95) cycling, 19% (n = 390) using public transport (train/bus) and 15% (n = 310) using private transport (car or other mode).

**Table 2:** **Characteristics of participating children**

**School**	**Age: mean (SD)**	**Gender: n (%) female**	**Ethnicity: n (%)**				*Usual mode of travel to school: n (%)			
			**White**	**Asian**	**Black**	**Other**	**Walk**	**Cycle**	**Train/bus**	**Car/private**
**L 1**	11.5 (0.6)	88 (58%)	123 (82%)	8 (5%)	11 (7%)	9 (6%)	93 (62%)	4 (3%)	53 (35%)	15 (10%)
**L 2**	11.6 (0.6)	0 (0%)	15 (11%)	101 (76%)	9 (6%)	10 (7%)	108 (80%)	4 (3%)	27 (2%)	9 (7%)
**L 3**	11.5 (0.6)	32 (37%)	66 (76%)	2 (2%)	8 (9%)	11 (13%)	32 (37%)	4 (5%)	43 (49%)	24 (28%)
**L 4**	11.5 (0.5)	100 (42%)	12 (5%)	11 (5%)	5 (2%)	209 (88%)	231 (97%)	0	7 (3%)	0
**L 5**	12.0 (0.2)	80 (53%)	11 (7%)	16 (11%)	8 (5%)	115 (77%)	136 (91%)	1 (0.7%)	7 (5%)	4 (3%)
**L 6**	11.6 (0.6)	215 (100%)	170 (79%)	10 (5%)	17 (8%)	18 (8%)	149 (69%)	2 (0.9%)	63 (29%)	41 (19%)
**L 7**	11.6 (0.5)	84 (49%)	127 (74%)	5 (3%)	23 (14%)	15 (9%)	76 (45%)	0	110 (65%)	24 (14%)
**Summary**	**Mean 11.6 (SD 0.2)**	**Total 599**	**Total 524**	**Total 153**	**Total 81**	**Total 387**	**Total 825**	**Total 15**	**Total 310**	**Total 117**
**R 1**	11.5 (0.5)	155 (55%)	234 (83%)	16 (6%)	6 (2%)	24 (9%)	255 (91%)	10 (4%)	4 (1%)	27 (10%)
**R 2**	11.3 (0.5)	67 (51%)	45 (34%)	8 (6%)	12 (8%)	66 (50%)	107 (82%)	10 (6%)	10 (6%)	19 (14%)
**R 3**	11.7 (0.6)	155 (100%)	59 (38%)	47 (31%)	33 (21%)	16 (10%)	101 (65%)	1 (0.6%)	46 (30%)	36 (23%)
**Summary**	**Mean 11.5 (SD 0.2)**	**Total 377**	**Total 338**	**Total 71**	**Total 51**	**Total 106**	**Total 463**	**Total 21**	**Total 60**	**Total 82**
**V 1**	10.2 (0.6)	120 (75%)	Missing data				163 (63%)	38 (15%)	17 (7%)	82 (32%)
**V 2**	11.5 (1.8)	49 (50%)	Missing data				64 (65%)	21 (21%)	3 (3%)	29 (30%)
**Summary**	**Mean 10.8 (SD 0.9)**	**Total 169**	**-**	**-**	**-**	**-**	**Total 227**	**Total 59**	**Total 20**	**Total 111**
**Overall** 2068	**11.5 (SD 0.7)**	**1145 (55%)**	**862 (50%)**	**224 (13%)**	**132 (8%)**	**493 (29%)**	**1515 (73%)**	**95 (5%)**	**390 (19%)**	**310 (15%)**

*Could chose more than one mode of travel; hence figures add up to more than 100%.

### Retention of children (objective 3)

**Figure 1:**
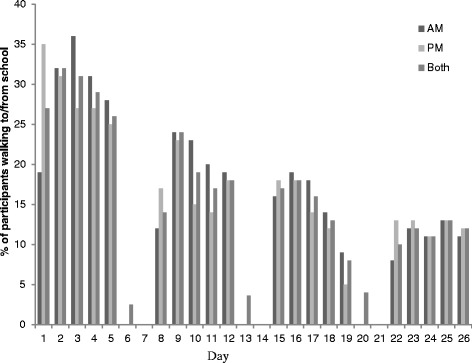
**Mean proportion of participants walking to/from school over the 4-week intervention period (across all schools).** *A walk was recorded when a participant scanned their card along at least 2 sensors going to or from school.

Of those who agreed to participate in the intervention, 54% (n = 2068) provided questionnaire data at baseline and 27% (n = 1025) immediately post-intervention. Data collected using the swipe cards (Figure 1) demonstrated that 100% of those registered to take part used their card at least once during the competition period. However, there was significant variation in participation consistency with 16% of children swiping their cards almost every day over the competition period and 35% using their card on five or fewer days. Differences were noted between countries, for example, 97% of participating children from Canada used their cards five times or more compared with 53% of children from England. Although there were some issues such as intermittent signal loss, the technology largely worked well for monitoring and recording walking behaviour.

### Intervention Effect (objective 4)

#### Walk to/from school

Figure 1 shows the mean number of children walking to and from school over the 4-week intervention period across all schools. Results show that on average 29% (SD 2.5) of children registered to participate walked to and from school in week 1. There was a gradual decline in the average number of children walking to and from school over the 4-week period (week 2 mean 18% ± SD3.6; week 3 mean 14% ± SD4.0; week 4 mean 12% ± SD1.1). Focus group discussions with children suggested that the reasons for the decline in walking may be due to a number of factors, for example, losing their “Beat the Street” swipecard, not having any sensors near their house or in their area, and living too far away to walk the whole way and thinking that the competition was therefore not relevant to them.

**Figure 2:**
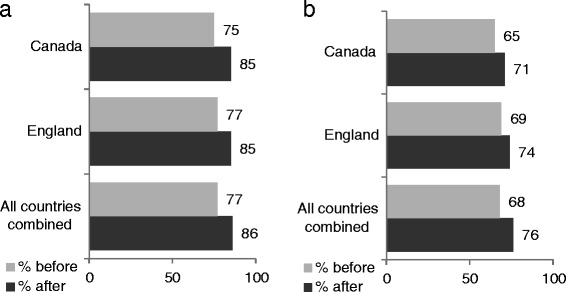
**Children’s walking behaviour (based on self-report data at baseline and post-intervention). a**. Children who walked at least once to or from school in the past week. **b**. Children who walked at least five to ten times to or from school in the past week.

Self-report data showed that at baseline, 77% (n = 601) of children stated they had walked to or from school at least once in the past week compared to 86% (n = 672) post-intervention (Figure 2a). Two thirds of children (68%/n = 531) said they walked on five or more journeys to or from school in the past week at baseline. Post-intervention, 76% (n = 594) of children stated they walked on at least half of the possible journeys to and from school in the past week (Figure 2b). Overall, 59% (n = 461) stated they walked more by the end of the competition period.

**Figure 3:**
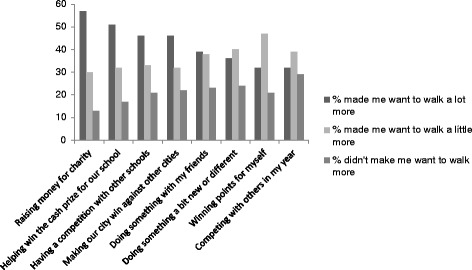
**Children’s views regarding the most important components of the intervention (based on self-report data post-intervention).**

Figure 3 highlights the main intervention components and reasons for children walking more during the intervention. The key factors included raising money for charity (57%), helping win the cash prize for our school (51%), having a competition with other schools (46%), and making their city win against other cities (46%).

Children further elaborated on the positive influence of the competition element in the focus groups, including:

- *“I find it very enjoyable and fun trying to collect the points. The competition is something different which makes it better.”* (girl from England, quote from focus group).
- *“It keeps you healthy and makes it more exciting to walk to school.”* (boy from England, quote from focus group).

#### Children’s attitudes

**Figure 4:**
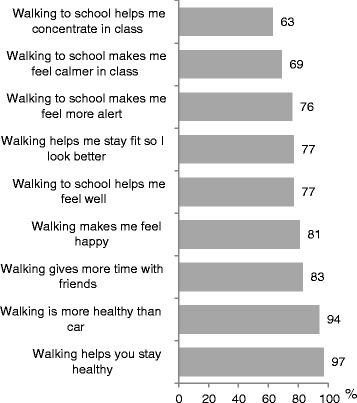
**Children’s attitudes about the benefits of walking (based on self-report data post-intervention).**

Figure 4 shows results for children’s attitudes towards walking. Post-intervention findings have been presented to ensure that all children had experience of regular walking in order to answer questions in regards to their attitudes towards walking. In total, 97% (n = 758) of children felt that walking to school helped them stay healthy, 81% (n = 633) felt happy and 76% (n = 594) helped them stay alert in class, 69% (n = 539) felt calmer and 63% (n = 492) more able to concentrate in class.

The majority of children (83%/n = 648) felt that walking to and from school let them spend more time with their friends (Figure 4). This was supported by data from the focus groups.

*“I came early and walked with my friends instead. We arranged to meet up and did that every day.”* (boy from Canada, quote from survey).

Further, 33% (n = 258) of children said that they had made new friends by walking to or from school during the competition.

*“Nearly everyone competing in Beat the Street has to admit that they at least said hi once to someone they didn’t know doing Beat the Street. This happened to me multiple times. Once I walked down a long road talking to a kid I didn’t know who I met through Beat the Street. Other kids approached me saying ‘oh you’re doing Beat the Street’ which encourages you more.”* (boy from England, quote from focus group).

Parents and teachers agreed with this finding; 91% of parents and 72% of teachers surveyed stated that they thought the competition had encouraged children to spend more time walking with their friends (data not shown). This was confirmed with data from the focus groups.

Using a check list, children also identified the following barriers to walking to school: poor weather (37%/n = 289), a perception that it took too long to walk (27%/n = 211) or that walking would make them late (23%/n = 180), and being driven by a family member (18%/n = 141).

Focus group data also suggested that children had positive reactions to participating in the intervention. Key motivating factors identified included having fun, enjoying the immediate gratification when their swipe card beeped on the scanners and enjoying time with friends. Children suggested that having a competition with their friends and classmates had motivated them to walk more. In addition, children said that raising money for charity was an important incentive. All of the children involved in the focus groups appeared enthusiastic about the competition and thought it should be run again in future.

*“I wanted to beat my friends and my cousin at another school. I walked to school most days, even when it rained which I would never have done before.”* (boy from England, quote from focus group).

#### Parent and teacher responses

Findings from interviews and online surveys demonstrated that having parents and teachers actively engaged in the process was a key facilitator. Parents and teachers thought it had potential to help children learn about schools in other countries, raise money for charity, reduce traffic congestion and increase children’s walking. Further, in locations where the intervention was implemented most successfully, there appeared to be a real partnership between schools, parents and local authorities. Teachers and parents at every school wanted the intervention to continue suggesting that the competition could provide a stimulus to encourage children to walk to school.

*“Encouragement and perks and pushing from school and parents helped get children out there. We did it together.”* (parent from Canada).

### Areas for programme modification and improvement (objective 5)

The following areas for modification and improvement were identified through the qualitative findings from children, parents and teachers:

- Running the intervention in Spring, as opposed to Autumn, would mean better weather and more lead-in time for preparation rather than beginning immediately after a new school year starts.
- Fitting registration and robust data collection methods into routine school processes was a challenge. There is a need to streamline the registration and data collection processes to make it as quick and easy for children and teachers to participate as possible, whilst ensuring that robust and appropriate methods are employed.
- Schools did not always seem clear about their roles and responsibilities throughout the study which included, for example, showing children where sensors were located and providing regular updates in form classes.
- Teachers felt that parental attitudes were a barrier for children walking to school, particularly in regards to safety and that future work was needed to address these negative perceptions.

## Discussion

Active travel can be an important contributor to children’s physical activity levels. However, previous interventions have shown modest effects at best and new approaches are required. This study investigated the feasibility of implementing a novel international walk to school competition involving schools from England and Canada, to increase active travel among children aged 9–13 years. To our knowledge this is the first study to incorporate an international element into such an intervention.

In total, 3817 children (representative of both genders and ethnic background) took part in the intervention from 12 schools in three cities (London, Reading and Vancouver) representing a 95% intervention participation rate for those invited. This demonstrates that the intervention has appeal and feasibility of recruitment of both schools and the children attending them. Results from several data sources including swipecard records, survey, interviews and focus groups with children, parents and teachers also provide preliminary evidence that the competition element increased children’s interest and engagement in walking to school.

However, findings from the objective tracking system showed a graded decline in the average number of children walking to and from school over the 4-week period. This decline in walking is suggestive of pro-innovation bias, explained by the Diffusion of Innovation Theory [29]. Diffusion is the process by which an innovation is communicated through certain channels over time among the participants and relies heavily on human capital. The innovation must be widely adopted and reach a critical mass in order to self-sustain. Results also suggest that the intervention requires modification to include further strategies to facilitate longer term behaviour maintenance. Further, 73% of children reported walking as their usual mode of travel to/from school at baseline which may explain the small change in walking behaviour post-intervention. This baseline figure is also higher than average walking to/from school levels which may be due to the intervention targeting city centre schools. Also, the majority of participating schools were based in London where the congestion charge may have played a key role in children having to walk at least some of the journey to/from school. Results suggested some discrepancy between walking levels collected using self-report questionnaires at baseline and the swipecard technology. Both over-reporting using self-report questionnaires and under-use of the swipecards were factors in explaining this discrepancy. Issues regarding social desirability and social approval have been well documented as limitations of self-reported measures of activity [30-32]. Further, children simply forgetting their swipecards, forgetting to swipe their card and walking different routes where sensors were not placed were all identified during focus group discussions as potential reasons to explain the under-use of swipecards.

### Recruitment

The current study used a pragmatic approach which involved collaborating with local authorities and transport organisations to identify schools that had a particular need to increase walking to school behaviour. This approach successfully recruited the target number of schools within the given timeframes from 12 schools in three different cities. Therefore the school recruitment strategies seem acceptable and appropriate for employment across different countries.

Recruitment of participants in physical activity trials are a commonly cited problem [33]. An intervention participation rate of 95% in a short time period, from 12 schools in two different countries is evidence of employment of a successful recruitment strategy for children. A key aspect of this success was due to the partnerships established with local authorities, school Principals, teachers and parents, and the innovative nature of the intervention (involving novel technology, bespoke website and incentives).

### Novel technology

The technology (swipecard and sensors) was a feasible element of the intervention as corroborated by the qualitative findings, swipecard data and minimal technical issues occurred throughout the 4-week intervention period. The high intervention participation rate and positive qualitative findings suggested that the novel technology played an integral role in the initiation of walk to school behaviour and the children were interested in the intervention. In particular, children from Vancouver had a higher retention rate which may have been due to greater support from the schools and parents. However, further modifications are required in order to ensure that walk to school behaviour is maintained throughout the 4-week intervention and beyond. Findings from the swipe card data suggest that participation in the competition and usage of the technology declined over the 4-week intervention period. This finding is similar to that reported in an earlier study involving similar technology [27]. Careful consideration needs to be given as to how compliance can be maintained over the intervention period in the next phase of the study. For example, the website could incorporate evidence-based behaviour change tools [27,34], and continued promotion from the school and project team. Findings suggest that the competition (extrinsic motivation) may act as a catalyst to stimulate walk to school behaviour. However, the intervention must incorporate other factors, such as social support [35], in order to sustain behaviour change long term.

### Internationalisation

Findings demonstrated that it was possible to simultaneously implement the intervention on an international scale. We postulated that the international element would add interest and stimulus to aid participation. Qualitative findings support this hypothesis as children suggested that competing against other international schools and finding out about other cultures was a key element in their decision to participate. However, further research is required to ascertain if the suggested benefits of internationalisation are worth the additional cost of implementation and evaluation of the intervention.

### Evaluation methods

Only 54% of those who participated in the intervention took part in the evaluation aspect and 27% provided follow-up data. Poor questionnaire completion rates at baseline and post-intervention follow-up suggest that the website (57% of responses were collected via the website) was not a suitable tool for data collection and that further consideration is needed in this regard before moving to the next stage of the trial. There is also a need to employ a validated, objective primary outcome measure of overall physical activity levels, for example, steps/day measured using a pedometer.

### Implications for future research

This current study provides an example of how partnerships with local authorities and organizations could be facilitated through the sponsorship of prizes and technology. Although the current study was funded through commercial sponsorship, for large scale, sustained roll out we envisage local authorities, for example, those with a remit for health (e.g. increasing physical activity) and transport (e.g. reducing traffic congestion and road safety incidents) will fund the scheme. Evidence from a similar scheme in Northern Ireland suggests that such a sustainable model is achievable [27]. However, such purported benefits need investigating in a larger trial. Following the MRC guidelines for the development and evaluation of complex public health interventions [36], the next stage of the study involves a larger controlled pilot trial and investigating whether purported benefits are maintained at longer term follow-up.

### Strengths and limitations

A particular strength of this study was the partnerships established with local authorities, transport organizations, school Principals, teachers and parents. This was highlighted as a key contributor towards the success of the trial. Further, partnership working facilitated a streamlined recruitment process and high intervention participation rate. As this is a feasibility study, no formal sample size calculation was conducted (as the information required is not available). However, we believe that recruitment of 3817 children was sufficient to meet the outlined study objectives. Data were collected across three different cities, each of which implemented the competition in slightly different ways, for example, in Canada there was greater parental and community involvement. However, these nuances in implementation demonstrate that it was feasible to implement the technology in different countries and cultures. Given the nature of school recruitment, there may be potential for selection bias as schools were mainly suggested by local authorities.

## Conclusions

This mixed methods feasibility study suggests that an intervention involving competition and innovative technology may be a novel way to motivate schools and children to initiate walking to school, which is supported by a high intervention participation rate. The results support the feasibility of the intervention and the use of novel technology in terms of its appeal to schools, children and their parents. The study also highlights the importance of partnership working as a key factor in the successful intervention implementation.

## Additional file

Additional file 1:
**Diagram depicting an example of the zonal rings at different distances that the sensors were placed around the school.**

## Abbreviations

CMO: Chief Medical Officer’s
Km: Kilometer
MRC: Medical Research Council
SPSS: Statistical Package for the Social Sciences
UK: United Kingdom
US: United States
