# Supplementary figures and images for: International inter-school competition to encourage children to walk to school: a mixed methods feasibility study
Source: BMC Res Notes. 2015 Jan 27;8:19. doi: 10.1186/s13104-014-0959-x (PMC4349778; doi:10.1186/s13104-014-0959-x)

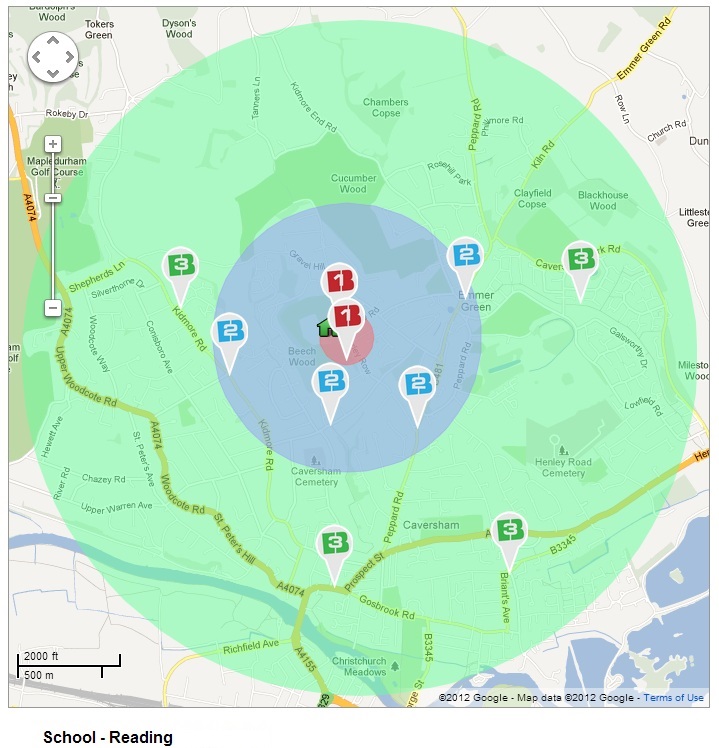

Supplement: Additional file 1: — Diagram depicting an example of the zonal rings at different distances that the sensors were placed around the school. [file 13104_2014_959_MOESM1_ESM.jpeg]
